# Multidetector computed tomography angiography of the celiac trunk and hepatic arterial system: normal anatomy and main variants *

**DOI:** 10.1590/0100-3984.2014.0041

**Published:** 2016

**Authors:** Severino Aires Araujo Neto, Carlos Fernando de Mello Júnior, Henrique Almeida Franca, Cláudia Martina Araújo Duarte, Rafael Farias Borges, Ana Guardiana Ximenes de Magalhães

**Affiliations:** 1 PdD, Associate Professor II of Medical Radiology at Universidade Federal da Paraíba (UFPB), João Pessoa, PB, Brazil.; 2 PhD, Associate Professor IV of Medical Radiology at Universidade Federal da Paraíba (UFPB), João Pessoa, PB, Brazil.; 3 Graduate Students of Medicine at Universidade Federal da Paraíba (UFPB), João Pessoa, PB, Brazil.

**Keywords:** Anatomy, Celiac trunk, Hepatic artery, Multidetector computed tomography

## Abstract

Although digital angiography remains as the gold standard for imaging the celiac arterial trunk and hepatic arteries, multidetector computed tomography in association with digital images processing by software resources represents a useful tool particularly attractive for its non invasiveness. Knowledge of normal anatomy as well as of its variations is helpful in images interpretation and to address surgical planning on a case-by-case basis. The present essay illustrates several types of anatomical variations of celiac trunk, hepatic artery and its main branches, by means of digitally reconstructed computed tomography images, correlating their prevalence in the population with surgical implications.

## Abstract (pt)

Embora a angiografia digital permaneça como padrão ouro no estudo do tronco celíaco e sistema arterial hepático, o exame por tomografia multidetectores associada às ferramentas informáticas de reconstrução de imagens digitais tem representado uma alternativa útil, principalmente por serem métodos não invasivos. O conhecimento detalhado tanto da anatomia normal quanto das variações anatômicas ajuda na interpretação de exames radiológicos e na adequação do planejamento cirúrgico para cada paciente. Este texto ilustra uma série de variações anatômicas do tronco celíaco e sistema arterial hepático, por meio de imagens tomográficas com reconstruções digitais, correlacionando as prevalências populacionais e implicações cirúrgicas.

## INTRODUCTION

Developments in surgical techniques such as upper abdominal videolaparoscopic surgeries, liver transplantation and gastrectomies, besides invasive and noninvasive procedures in the abdomen, require of the health professional a wide knowledge about the anatomy of the celiac arterial trunk (CAT), hepatic arterial system (HAS) and their main variations^(1-4)^ .

Angiography is the gold standard for CAT, and its branches, visualization^(1)^. However, with the arrival of multidetector computed tomography (CT) angiography and modern image reconstruction programs, this imaging method becomes an additional option for the detailed investigation of arteries with the significant advantage of its noninvasiveness^(5)^. Multidetector CT angiography allows the visualization of small caliber short arteries by means of maximum intensity projection (MIP) and three-dimensional volume rendering (VR) techniques.

The present essay is aimed at describing the normal anatomy and commonly found CAT and HAS variations.

The images shown in the present essay were collected from the personal files of the authors and acquired in a Brilliance 64-channel multidetector CT apparatus (Philips Medical System; Best, The Netherlands).

The scan protocol, with small sporadic variations, consisted in axial sections, slice thickness of 1 mm, pitch 0.8. The contrast agent Ultravist (Bayer) was utilized, at a concentration of 769 mg/mL, intravenously injected by means of an injection pump at a rate of 5 mL/s, with bolus tracking time delay. A standard field of view (250 mm) was utilized. The images reconstruction thickness was 2 mm. An Extended Brilliance Work Space workstation was utilized with the software Philips Brilliance for computed tomography (Philips Medical Systems; Best, The Netherlands).

 In order to define the arterial pattern, analyses were performed in the axial plane with reconstruction techniques in the coronal and sagittal planes in multiplanar reconstructions (MPR), as well as three-dimensional reconstructions with the MIP and VR techniques. The normal patter and the main CAT and HAS variations were demonstrated.

## CELIAC ARTERIAL TRUNK AND HEPATIC ARTERIAL SYSTEM: NORMAL ANATOMY AND VARIATIONS

CAT variations are not infrequent^(1)^. Song et al. have studied 5,002 cases of CAT and reported the occurence of variations in 10.9% of cases^(5)^. However, as concomitant CAT and HAS variations are considered, the rate increases to 55% of patients^(3)^ .

**Figure 1:**
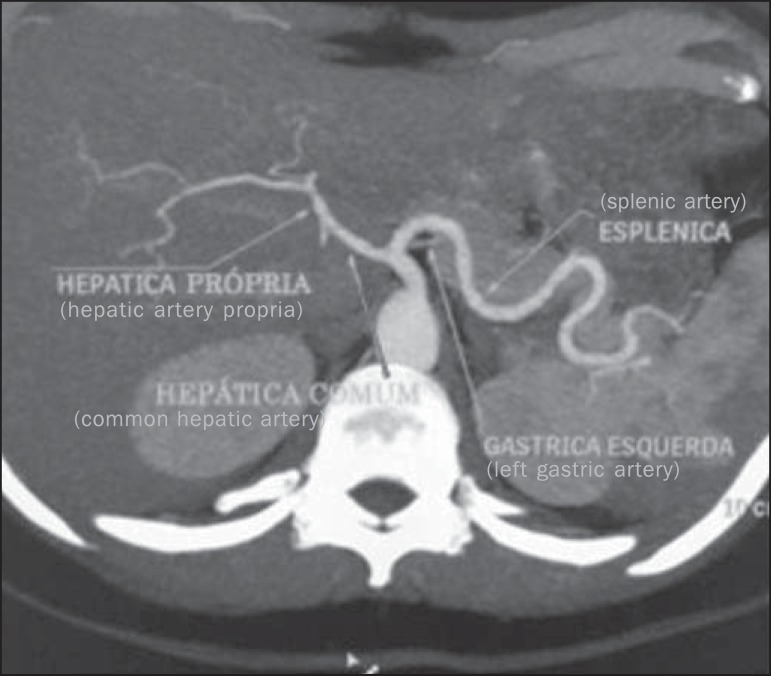
Contrast-enhanced axial CT demonstrating a normal CAT. The celiac artery trunk represents an arterial triad consisting of the left gastric artery, common hepatic artery and splenic artery indicated by the arrows.

The normal celiac trunk - 89.1% of cases^(5)^ - is described as the trifurcation originating the left gastric artery, splenic artery and common hepatic artery^(3,5)^ (Figure 1). Normally, the left gastric artery is the first branch of the CAT and runs cranially toward the smaller curvature of the stomach were it undergoes anastomosis with the right gastric artery; the splenic artery is the branch of the trunk with largest caliber and runs tortuously toward the spleen; the common hepatic artery runs to the right where it divides into gastroduodenal artery inferiorly, and hepatic artery propria superiorly^(2,4)^ .

**Figure 2:**
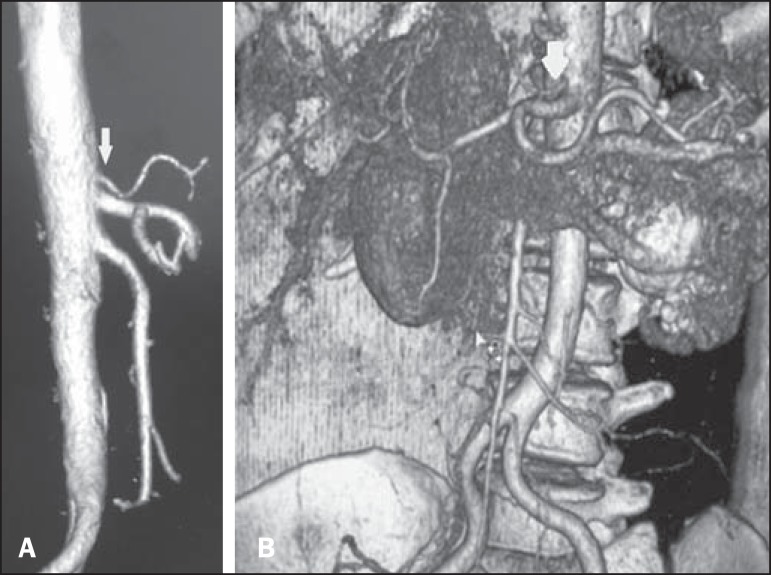
Computed tomography, coronal section with VR, demonstrating a hepatosplenic trunk. The arrow indicates the left gastric artery emerging from the aorta, above the hepatosplenic trunk.

**Figure 3:**
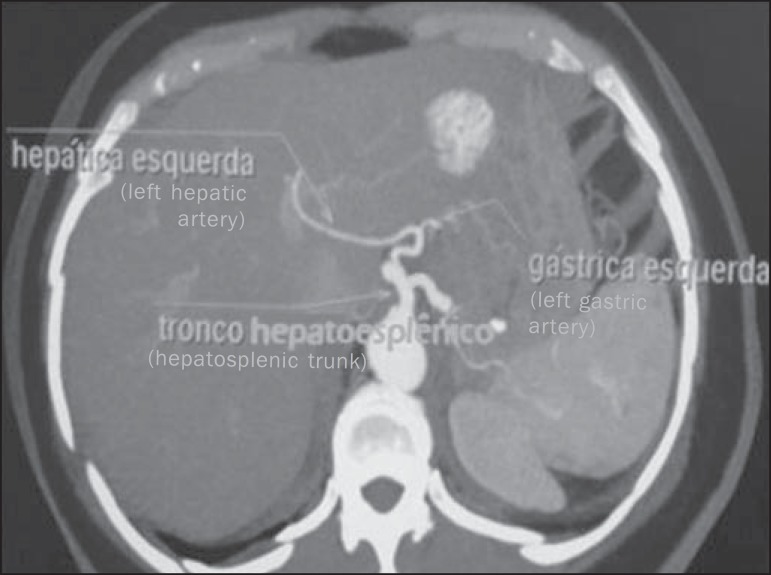
Contrast-enhanced axial computed tomography. The image demonstrates a case of hepatosplenic trunk with left gastic artery relocation, so in this case it emerges from the left hepatic artery.

**Figure 4:**
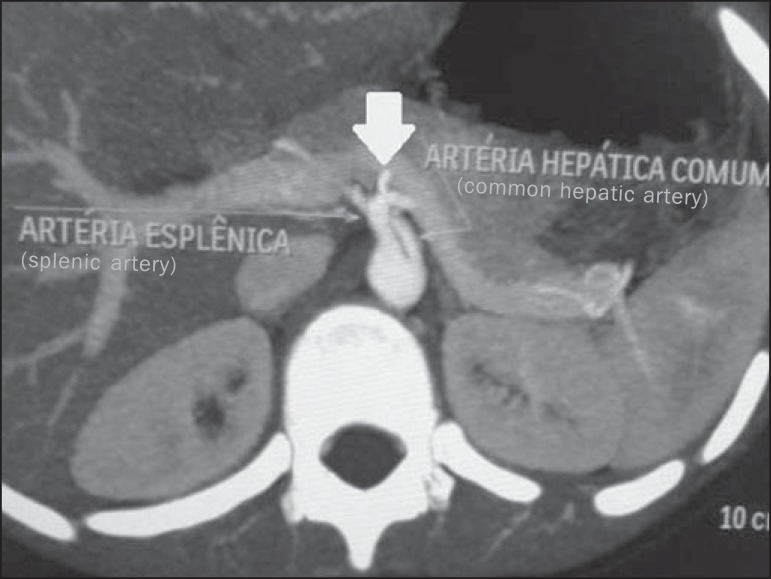
Contrast-enhanced axial computed tomography showing a splenogastic trunk (consisting of splenic artery and left gastric artery – indicated by the arrow). In this case, the common hepatic artery is a branch from the aorta.

**Figure 5:**
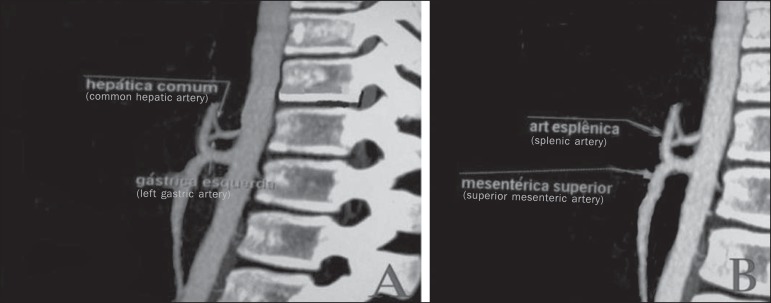
Contrast-enhanced sagittal computed tomography. **A:** The image shows a case of hepatogastric trunk. The arrows indicate the arteries composing this trunk (common hepatic artery and left gastric artery). **B:** The image shows that the splenic artery, in this case, emerges from a common trunk with the superior mesenteric artery.

The most common CAT variations are the following: hepatosplenic trunk, representing about 3% of cases, where the common hepatic artery and the splenic artery originate from a single trunk, and the left gastric artery is located above this trunk, either in the aorta or in other artery of the upper abdomen (Figures 2 and 3)^(6)^; splenogastric trunk (4%), where the left gastric artery originates from the splenic artery, forming a common trunk (Figure 4); hepatogastric trunk (1%), with the left gastric artery and common hepatic artery originating from a single trunk (Figure 5). The absence of the CAT is rarely described in the literature (0.1%)^(5)^ .

As regards the HAS, it is described as normal in cases where the common hepatic artery originates the hepatic artery propria after the emergence of the gastroduodenal artery; and the hepatic artery propria, on its turn, divides into right and left hepatic arteries within the hepatoduodenal ligament, at few centimeters from the liver surface.

**Figure 6:**
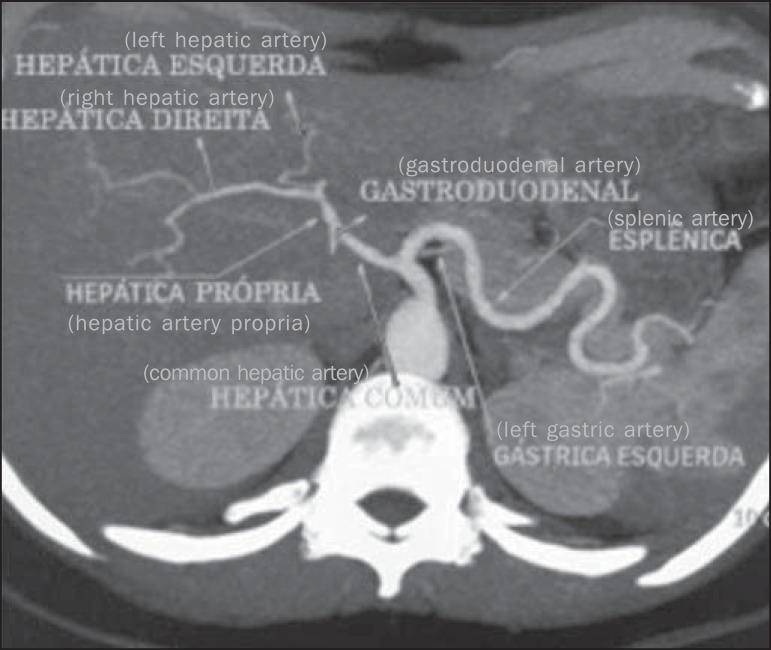
Contrast-enhanced axial computed tomography. The image presents a case of normal pattern of the HAS, with the hepatic artery propria originating from the common hepatic artery, after the emergnce of the gastroduodenal artery; and right and left hepatic arteries emerging from the hepatic artery propria (Hiatt’s type I).

According to Koops et al., the frequencies of the normal HAS pattern as per the Hiatt's classification (Table 1), are contained in the interval 59-79.1% (type I) (Figure 6).

**Table 1:** Anatomical variations of the hepatic artery according to Hiatt's classification

Anatomical variation of the hepatic artery	Hiatt's classification
Normal anatomy	Type I
Left hepatic artery or accessory left hepatic artery relocation	Type II
Right hepatic artery or accessory right hepatic artery relocation	Type III
Left hepatic artery or accessory left hepatic artery relocation and right hepatic artery or accessory right hepatic artery relocation	Type IV
Common hepatic artery originating from superior mesenteric artery	Type V
Common hepatic artery originating from the aorta	Type VI

**Figure 7:**
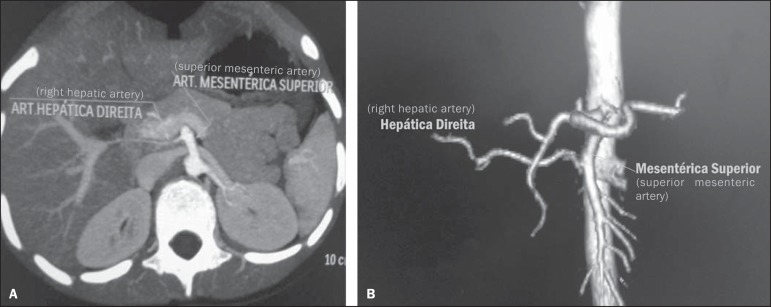
The image presents a case of right hepatic artery relocation, where it emerges from the superior mesenteric artery (Hiatt’s type III). Contrast-enhanced axial computed tomography (**A**) and contrast-enhanced computed tomography with VR reconstruction (**B**) showing right hepatic artery relocation.

Amongst the most described variations, the following frequencies were found: 3-17% (type II), relocation of the left hepatic artery; 7-18% (type III), relocation of the right hepatic artery (Figure 7); and 1.5-5% (type V), common hepatic artery originating from the superior mesenteric artery. Also, according to Hiatt, it is possible to find non-classified variations with a frequency of 1-4.1%^(7)^ .

Normally, cases of Hiatt's type III are the most prevalent and play a relevant role in procedures involving the liver, as after originating from the superior mesenteric artery, the right hepatic artery runs posteriorly to the portal vein, which might confuse the surgeon, since in the normal pattern (type I) such artery is located anteriorly to the portal vein, within the hepatoduodenal ligament. Thus, one of the reasons for understanding those variations is avoiding iatrogenic events^(8)^.

In Hiatt's type II - left hepatic artery relocation -, procedures such as gastrectomy should be cautiously performed, considering that in most of such cases, the left hepatic artery emerges from the left hepatic artery; thus, in case of section of the left gastric artery, a possible ischemia of the whole functional left hepatic lobe might occur.

Understanding the pattern of variation of the hepatic arteries becomes imprescindible for the development of the liver transplant^(9)^.

With the introduction of laparoscopic surgeries with reduction of the surgical field view, it is necessary to understand the pattern of variations of the upper abdomen^(10)^.

The arterial patterns are relevant in the planning of the whole surgical and radiological procedure involving the upper abdomen^(5)^.

Considering the relevance of the mentioned variations, the authors suggest that radiologists should investigate the arterial pattern and inform them in reports of surgeries and invasive examinations of the upper abdomen.

## CONCLUSION

Considering that the vascularization of a great part of the gastrointestinal system occurs from CAT and HAS branches, the knowledge about anatomical variations and respective frequencies is of paramount relevance in the planning of upper abdomen surgeries to avoid procedural errors and medical iatrogenic events.
